# Hepatocellular Carcinoma: Causes, Mechanism of Progression and Biomarkers

**DOI:** 10.2174/2213988501812010009

**Published:** 2018-06-29

**Authors:** Amit Kumar Singh, Ramesh Kumar, Abhay K. Pandey

**Affiliations:** Department of Biochemistry, University of Allahabad, Allahabad 211002, India

**Keywords:** HCC, HBV, Hepatocarcinogenesis, Apoptosis, Biomarkers, MiRNA

## Abstract

Hepatocellular Carcinoma (HCC) is one of the most common malignant tumours in the world. It is a heterogeneous group of a tumour that vary in risk factor and genetic and epigenetic alteration event. Mortality due to HCC in last fifteen years has increased. Multiple factors including viruses, chemicals, and inborn and acquired metabolic diseases are responsible for its development. HCC is closely associated with hepatitis B virus, and at least in some regions of the world with hepatitis C virus. Liver injury caused by viral factor affects many cellular processes such as cell signalling, apoptosis, transcription, DNA repair which in turn induce important effects on cell survival, growth, transformation and maintenance. Molecular mechanisms of hepatocellular carcinogenesis may vary depending on different factors and this is probably why a large set of mechanisms have been associated with these tumours. Various biomarkers including α-fetoprotein, des-γ-carboxyprothrombin, glypican-3, golgi protein-73, squamous cell carcinoma antigen, circulating miRNAs and altered DNA methylation pattern have shown diagnostic significance. This review article covers up key molecular pathway alterations, biomarkers for diagnosis of HCC, anti-HCC drugs and relevance of key molecule/pathway/receptor as a drug target.

## 1 INTRODUCTION

Liver cancer is one of the leading causes of cancer deaths worldwide. In recent years, the annual death toll with 700,000 has been recorded around the globe [1]. Hepatocellular Carcinoma (HCC) is the major form of liver cancer. Risk factors for HCC include chronic HBV (hepatitis B virus) and HCV (hepatitis C virus) infections, autoimmune hepatitis, chronic alcohol use, obesity and diabetes mellitus *etc* [2]. Between 1990 and 2013, about 63% increase in total deaths has been reported globally because of viral hepatitis. Hepatitis B and C infections accounted for most of the morbidity and mortality since it leads to progressive hepatic damage in patients and ultimately causing cirrhosis and hepatocellular carcinoma [3].

In areas of high incidence, HCC has been reported in children of even two years of age. However, the incidence increases with age in all populations and shows a slight decline in the elderly population. HCC shows a strong male preference. In low incidence regions, it is four times more common in males while in high prevalence areas, it is about eight times more common. This report may be partially ascribed to the collective effect of other factors including higher levels of alcohol intake and smoking coupled with a higher incidence of cirrhosis in males. Animal experiments have suggested the role of sex hormones and/or hormone receptors. Orchidectomy reduces the carcinogenic effects of chemicals in male rats to the level found in females. A similar effect has been observed with stilbesterol or estradiol pellets’ implantation but the effect was comparatively less [4].

In western countries, inborn errors of metabolism and congenital abnormalities have also contributed towards HCC in some cases [5]. The current review describes the varied causes, molecular mechanism, biomarkers and drug targets for the diagnosis and prognosis of hepatocellular carcinoma.

## 2 GENETIC AND CONGENITAL ABNORMALITIES

Inbred strains of mice have shown genetic susceptibility to cirrhosis and liver cancer. However, in man, it has not been documented. Chinese and Alaskan inhabitants display familial clustering of HCC [6, 7]. The occurrence of HCC is rarely reported in congenital hepatic fibrosis, ataxia telangiectasia, familial polyposis coli, familial cholestatic cirrhosis, fetal alcohol syndrome, situs inversus and neurofibromatosis [7]. Hereditary tyrosinemia, an inborn error of metabolism, is associated with the maximum risk of liver carcinoma [8]. Within a short span of time, these patients exhibited faster development of macro-nodular cirrhosis from micronodular cirrhosis, followed by dysplasia and finally HCC. Adenomas may be associated with type I glycogen storage disease but the occurrence of carcinoma is rare. Carcinogenic properties have been attributed to iron through free radical production [9]. An autosomal recessive disorder, Wilson’s disease, has a tendency to affect male population usually and causes cirrhosis *via *copper build up in the hepatic cells. Deficiency of alpha-1-antitrypsin, a protease inhibitor, is related to jaundice and cirrhosis during infancy, as well as with pulmonary emphysema and cirrhosis in adults [10].

## 3 HEPATITIS VIRUS

**Fig. (1):**
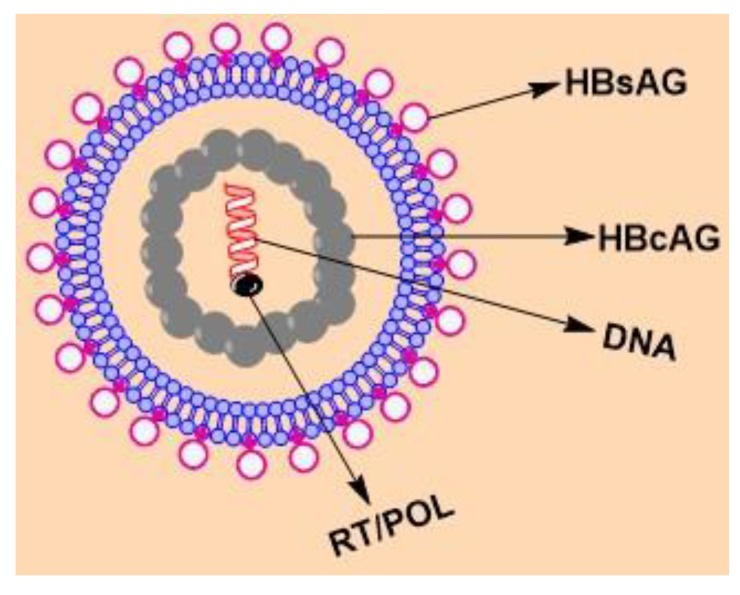
Structure of HBV.

The hepatitis viruses are unrelated human pathogens and are referred to as types A, B, C, D and E. HCC is one among ten most widespread cancers globally, is strongly related with HBV, and in some regions with HCV. HBV is a small encapsulated DNA virus having unusual reverse transcriptase activity [11, 12]. It belongs to family hepadnaviridae and has eight genotypes, A to H which have separate geographic distribution. It contains four overlapping transcription units encoding the nucleocapsid or core proteins consisting of the hepatitis B core antigen (HBcAg), the envelope proteins consisting of the Hepatitis B surface Antigen (HBsAg), the polymerase and the X protein (HBx) which has transcriptional trans-activating potential. The infectious viral particle, also known as Dane particle, is a spherical, double walled structure (diameter 42 nm) having a lipid envelope with HBsAg surrounding an inner nucleocapsid consisting of hepatitis B core antigen (HBcAg) complexed with a virally encoded polymerase and the viral DNA (Fig. **1**) HBV genome is 3.2 kb in size and made up of a partially double-stranded circular DNA. The 5′ terminus of the minus strand is covalently attached to the viral polymerase.

Hepatitis C Virus (HCV) is also a member of hepadnaviridae family. It contains a positive, single-stranded RNA genome having two untranslated regions at the 5' and 3' ends, and a large open reading frame encoding for a 3,010 to 3,030 amino acid polyprotein [13].

### 3.1 Mode of Transmission and Replication Cycle

**Fig. (2):**
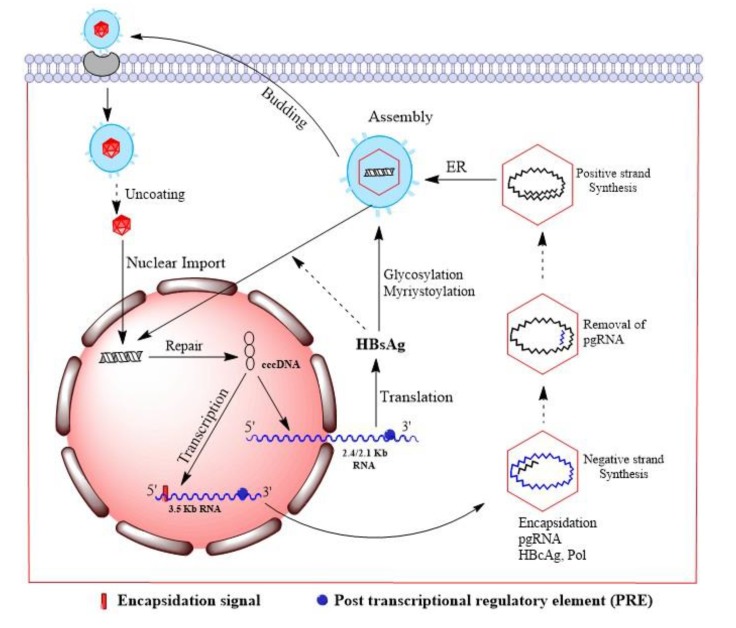
Replication cycle of HBV.

Contaminated food or water acts as a source for spreading Hepatitis A and E. Transmission of hepatitis B, C and D generally takes place through the infected body fluids. These viruses are usually transmitted through transfusion of infected blood, use of contaminated equipment during surgery, and sexual contact. HBV is also transmitted from mother to child during parturition. Acute infection may be symptomatic or non-symptomatic. Symptoms include yellow colouration of eyes, skin and urine, intense weakness, abdominal pain, nausea, and vomiting [14]. The HBV infection involves an initial step that is attachments of mature virion to the host cell surface, with the help of preS domain of the surface protein [15]. Various factors have been suggested to act as receptors for viruses in the cell. However, only carboxypeptidase D mediated viral entry has been revealed during duck HBV infection [16]. Disassembly of the virus and mechanism of intracellular nuclear transport for the viral genome are not clearly understood and nucleocapsid core protein modification has been implicated in the process [17]. After nuclear import viral DNA is converted to the covalently closed circular DNA (cccDNA) (Fig. **2**) [18]. The cccDNA transcripts do not undergo splicing and have a polyadenylated structure with a 5' cap. Two different 5' ends are present in the genomic transcripts (3.5kb) which consist of two species *i.e.*, the pregenomic RNA (pgRNA) and the precore RNA. The pgRNA serves as messenger RNA for core and polymerase as well as the template for reverse transcription. The pre-core RNA is translated into the pre-core gene products. Through ribosomal scanning mechanism of the pgRNA, the pol start codon initiates the polymerase translation [19]. The 2.4kb subgenomic RNA produces large HBsAg protein, 2.1kb RNAs produce the middle HBsAg (M-HBsAg) and small HBsAg (S-HBsAg) proteins, and 0.7kb RNA is translated to the HBxAg protein.

## 4 MOLECULAR MECHANISM OF HEPATOCELLULAR CARCINOMA

HCC is the outcome of many variable etiological factors such as HBV, HCV, alcohol, aflatoxins, inborn and acquired metabolic diseases. The carcinoma might originate in mature liver cells or progenitor cell. Hence, the molecular basis of HCC progression may differ depending on diverse factors and therefore, a number of mechanisms might be involved [20]. Some important mechanisms associated with the hepatocellular carcinogenesis are described below.

### 4.1 Loss of Cell Cycle Control

**Fig. (3):**
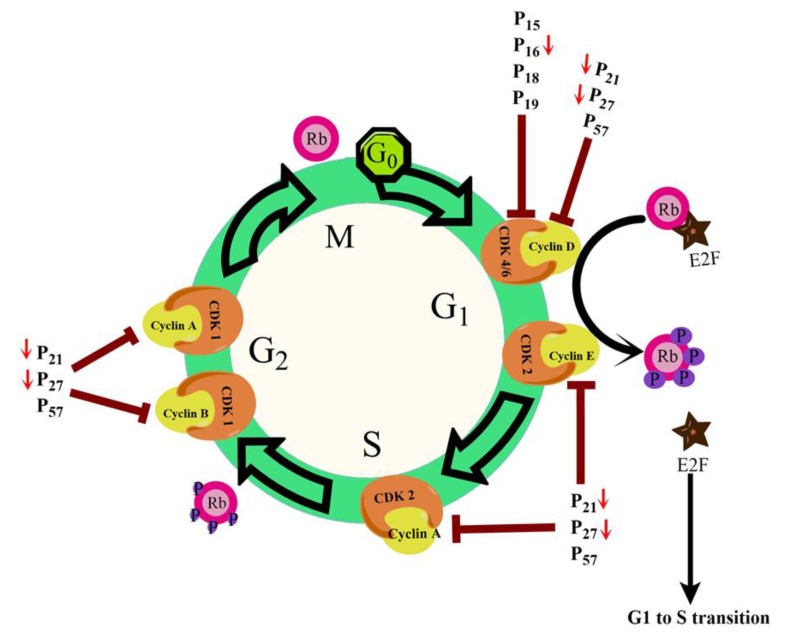
Cell cycle pathway and common checkpoints. The downward arrows indicate decreased expression of Ink4 family of Cdk inhibitor (p16) and Cip/Kinase inhibitory protein (Kip) family inhibitor (p21/27) during hepatocarcinogenesis. Release of E2F transcription factor leads to G1 to S transition.

Loss of cell cycle control is a general feature observed in all cancerous cells. This leads to an increased multiplicative tendency, hyperplasia, and subsequent tumour formation. Normal liver cells primarily live in the G0 phase (quiescent phase) of the cell cycle and renewed slowly. However, they possess the strong regenerative ability and after getting mitogenic signals, they enter the cell cycle and proceed to cell division [21]. Advancement through the eukaryotic cell cycle phases is governed by the combined actions of cyclins and cyclin-dependent kinases (Cdk). Cytokines and growth factors promote de-novo expression of cyclin D1 gene which is responsible for the transition of quiescent hepatocytes into the cell cycle [22, 23]. Many regulatory checkpoints apply the brake on free proliferation and avert quiescent hepatocytes from entry in the cell cycle (Fig. **3**). As depicted in Fig. (**3**), retinoblastoma (pRb) and other proteins bind to and seize E2F transcription factors and thereby repress its activity [24]. Entry into the cell cycle is also prevented by the Ink4 family of Cdk inhibitors (p15/16/18/19) by binding to Cdk4/6 kinases and inhibiting the formation of cyclin D-Cdk4/6 complex [25]. Binding of CDK interacting protein (Cip)/Kinase inhibitory protein (Kip)family inhibitory proteins p21/27/57 with Cdk/cyclin complexes inactivates it and inhibits cell cycle advancement [26]. A “proliferation cluster” has been identified in gene expression profiles of HCC samples which accounted for increased expression of proliferation-associated genes [27, 28]. Abnormalities that decrease the expression levels of p16/pRb genes or hamper their protein functions eventually cause tumorigenesis because p16/pRb pathway manages entry into cell cycle. Altered expression of the pRb is a universal phenomenon in HCC [29]. It has been reported that the expression levels of CIP/KIP family member proteins p21/27 are frequently reduced in HCC samples [30].

### 4.2 Loss of Senescence Control

**Fig. (4):**
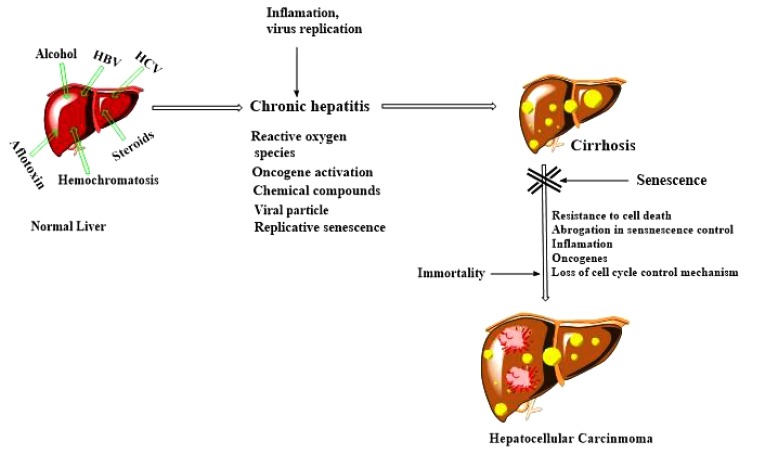
The proposed model of hepatocellular carcinoma development.

Senescence is a type of irreversible growth inhibition of cells in cell culture showing distinct morphological alterations [31]. In hepatocytes, mechanism of senescence is not clearly understood. Replicative senescence controls partial proliferative ability of liver cells by a gradual decrease in the telomeric segment [32]. Telomere-independent mechanisms have also been suggested for hepatocyte senescence monitored in severe chronic liver diseases and these include free radical and oncogene-dependent senescence The resulting DNA damage activates ATM/Chk/p53 pathway and arrests cells at G1 phase. Alternatively, the p16/pRb pathway also performs the same function. Anomalies in DNA damage checkpoint and cell cycle regulatory pathway paved a way for the unlimited proliferation of genetically altered hepatic cells at the senescent phase and subsequently to malignant transformation. (Fig. **4**).

In human HCC, the p53 pathway has an effect on many levels *i.e.*, (a) about 50% aflatoxin-mediated HCC cases exhibit p53 mutations while 20–30% cases of non-aflatoxin mediated HCC show p53 mutations; (b) microdeletions of p14^ARF^ rarely occurs in HCC with p53 mutation while it is reported in 15-20% of human HCC; (c) human HCC also shows elevated Mdm2 expression; (d) over expression of gankyrin, an oncoprotein, is commonly observed in human HCC, which imposes restriction on the pRb and p53 [33].

pRb pathway anomalies (p16, p15 or RB1 genes) are observed in more than 80% of human HCC. The anomalies include p16/15 promoter methylation and deletion or mutation of RB1 gene. Promoter methylation causing p16 repression is the most common anomaly [34]. Telomerase activation occurs during the transformation of precancerous lesions to HCC. Telomere-dependent senescence arrest in hepatocytes is frequently observed in cirrhosis. Reactivation of Telomerase Reverse Transcriptase (TERT) acts as a bypass for HCC growth. TERT is absent in normal hepatocytes, hence 90% human HCC show telomerase activation, a rate-limiting step for the commencement of cell immortality [35]. Deregulation of TERT expression by integration of HBV DNA into TERT gene is a rare phenomenon [29]. Besides, HBV surface proteins (viral X and PreS2) and HCV core protein may increase the activity of telomerase [36]. The above-mentioned facts indicate the cooperation between the anomalies in telomerase activity and senescence controlling genes (p53) during the hepatocarcinogenesis.

### 4.3 Dysregulation of Apoptosis

Cell death resulting from liver injury may be either accidental (necrotic), programmed (apoptotic), or uncontrolled. Extrinsic or intrinsic pathways initiate apoptosis by activating caspases 3, 6 and 7 [22, 37]. Death receptors mediate resistance towards apoptosis in HCC cells. The majority of the HCCs show one or more alterations in the Fas pathway molecules, which inhibit Fas-mediated apoptosis. HCC cells or tissues become unresponsive to Fas by downregulating Fas expression resulting in reduced expression of FADD or FLICE or increased expression of cellular FLICE/caspase-8-inhibitory protein (cFLIP), or by upregulation of nuclear factor-kappa B (NF-κB), Bcl-2 or Bcl-XL and Mcl-1 [38-40]. Pro-apoptotic proteins (Bax or Bcl-XS) are downregulated in HCC. The TGF-β pathway is regularly stimulated at the cirrhosis stage and promotes apoptosis by activating Smad3 mediated Bcl2 downregulation and thereby reducing the susceptibility towards HCC development [41]. Insulin-receptor signalling and activation of the PI3K-Akt pathway might also be involved in resistance towards apoptosis [42]. The insulin-like growth factor 2 receptor (IGF2R) reduces cell division by stimulating TGF-β signalling and breakdown of the IGF2 mitogen [43]. During the initial phase of human hepatocarcinogenesis heterozygosity in IGF2R locus is frequently lost [44]. In human HCCs loss of IGF2R and overexpression of IGF2 growth factor are common features. Stimulation of the Akt signalling and reduced expression of a negative regulator of Akt *i.e.*, phosphatase and tensin homolog (PTEN) have been described in 40-60% HCC cases [45].

### 4.4 Liver Inflammation and Hepatocarcinogenesis

Most of the studies suggest that liver injury in viral hepatitis does not result from the direct cytopathic effects of viruses but caused by the viral protein-mediated host immune response [46]. Animal studies have provided ample proof that viral hepatitis is triggered by an antigen-specific intrahepatic cellular response that set in motion a series of antigen-nonspecific cellular and molecular effector systems. Cellular and humoral limbs of the immune system work towards viral clearance by three different mechanisms: firstly, the virus-specific T-cell mediated direct destruction of infected hepatocytes; secondly, the removal of free viral particles from the circulation by the antibody response; and thirdly, non-cytopathic viral inactivation in infected hepatocytes by some inflammatory cytokines produced by activated mononuclear cells [47]. Recent evidence suggests that NF-κB signalling mediated inflammation plays an essential role in commencement, promotion and development of tumours [48].

#### 4.4.1 Cytokines

Various inflammatory cytokines viz., interleukin-1α (IL-1α), IL-1β, IL-6, IL-8 and tumour necrosis factor-α (TNF-α), participate in chronic hepatic inflammation. Among these, IL-6 is the most important and is produced by activated kupffer cells in chronic hepatitis. It results in local inflammatory response and activates hepatocyte proliferation leading to cancerous hepatocytes [49]. In chronic liver diseases such as HBV and HCV induced hepatitis, alcoholic hepatitis and non-alcoholic steatohepatitis increased serum IL-6 levels have been observed. These reports highlight the vital role played by IL-6 in human hepatocarcinogenesis. IL-6 knockout mice exhibited a significant reduction of diethylnitrosamine (DENA)-initiated HCC development, suggesting a direct involvement of IL-6 signalling in experimental hepatocarcinogenesis. Role of innate immune response in the hepatocarcinogenesis has also been demonstrated by IL-6 production *via *stimulation of Toll-Like Receptor (TLR) mediated through MyD88in rodents [50].

#### 4.4.2 **NF-**κ**B Pathway**

NF-κB, a transcription factor, plays a key role in innate immunity and liver inflammatory signalling pathways [51, 52]. It is activated by cytokines or interleukins such as TNF-α, IL-6 and IL-1β, viral and bacterial DNA and RNA and pathogen-derived lipopolysaccharides. NF-κB dimer formed after activation undergoes nuclear translocation, attaches with particular DNA segment, and triggers transcription of genes related to immune responses, inflammation, proliferation and survival of cells [53, 54]. In all chronic liver diseases viz., alcoholic/non-alcoholic/biliary liver disease and viral hepatitis NF-κB gets activated [55]. It has been demonstrated that inducible IκB super-repressor mediated NF-κB inhibition reduced hepatic tumour development in chronic inflammation induced Mdr2 knockout mouse, the animal HCC model [56, 57]. The liver tumour-promoting activity of NF-κB has been validated in another inflammatory HCC model *i.e.*, hepatocyte-specific lymphotoxin αβ transgenic mouse model. In this model, NF-κB was inhibited by hepatocyte-specific deletion of IKK-β which resulted in entirely reduced HCC progression [58].

## 5 Characterisation of Hepatocellular Carcinoma: Biomarkers

With the vast input of knowledge about tumour biology, curiosity for identifying HCC related molecular markers has increased. During the new era of “omics”, the emergence of a number of cutting-edge technologies such as next-generation sequencing and microarray has advanced the search for biomarkers [59-61]. These technologies have given an advantage in examining the tumour genome (single nucleotide polymorphism, variations in copy number, aneuploidy and loss of heterogeneity), transcriptome, proteome, epigenome, metabolome, and miRNA profile [62-64]. Currently, several markers in blood and tissue have been identified [65, 66]. A detailed account of various HCC markers is given below.

### 5.1 Metabolic Biomarkers

#### 5.1.1 α-Fetoprotein

Since the discovery of α-fetoprotein (AFP) in the serum of HCC patients, AFP is considered as the most important biomarker for assessment of HCC [67]. It is a glycoprotein (MW 70 kDa) responsible for transport of several compounds viz., steroids, bilirubin, retinoid, fatty acids, flavonoids, heavy metals, dioxin, dyes, phytoestrogens, drugs *etc* [68]. It is produced by the fetal liver, yolk sac and intestine during development [69]. During 12-16 weeks of fetal development, AFP in serum reaches the highest concentration (3 g/L). Subsequently, there is a rapid decline in the levels and only traces are detectable in serum [70]. Unusually elevated serum AFP levels find a correlation with the malignant diseases including HCC [71, 72]. AFP is found in three glycoforms based on lectin binding pattern *i.e.*, the non-binding fraction AFP-L1, the weak binding fraction AFP-L2, and the binding fraction AFP-L3. Liver cirrhosis and chronic hepatitis show elevated levels of AFP-L1, whereas in HCC AFP-L3 is notably increased. Only cancer cells produce AFP-L3 hence, it is regarded as specific HCC biomarker [73, 74].

#### 5.1.2 Des-γ-Carboxyprothrombin (DCP)

Des-γ-carboxyprothrombin (DCP) is an abnormal form of prothrombin and also called prothrombin induced by vitamin K absence-II (PIVKA II). The production of DCP stems from a defective vitamin K-dependent posttranslational carboxylation system, which induces the malignant transformation of HCC cells [75]. Normal prothrombin function of DCP is lost and it supports malignant growth in HCC. Serum DCP levels in patients (benign and malignant liver diseases) varies considerably. Its sensitivity as a diagnostic agent might be better than AFP. This result still needs validation [76].

#### 5.1.3 Glypican-3

Glypican-3 (GPC3) belongs to the glypican family of heparan sulfate proteoglycans. Glycosyl-phosphatidylinositol anchor links it to the cell membrane [77]. GPC3 is responsible for cell proliferation, survival, and tumour suppression, but is normally absent in healthy and non-malignant hepatocytes. GPC3 acts as a biomarker for different types of cancers. It is upregulated in HCC whereas it is downregulated in lung adenocarcinoma, ovarian cancer, and breast cancer [78, 79]. In HCC, it has been suggested to act as growth stimulator by upregulating autocrine/paracrine canonical Wnt signalling [80].

#### 5.1.4 Golgi Protein-73 (GP73)

GP73 (MW 73kDa) is present in the Golgi complex as a transmembrane glycoprotein. It is expressed in normal biliary epithelial cells whereas it is not expressed in normal hepatocytes. In hepatic diseases such as HCC, its expression is considerably enhanced [81]. It has been reported that serum GP73 in HCC patients was appreciably greater than in normal healthy persons and HBV carriers [82].

#### 5.1.5 Squamous Cell Carcinoma Antigen (SCCA)

Squamous Cell Carcinoma Antigen (SCCA) belongs to the family of serine protease inhibitors found in squamous epithelium and in cervical carcinoma. Epithelial tumours exhibit higher expression of SCCA and act as an anti-apoptotic agent [83]. Dedifferentiation results in SCCA expression and it is considered as a prospective HCC biomarker. It has been reported that HCC patients showed higher serum SCCA levels than patients with cirrhosis [84]. An alternative prospective marker is the SCCA complexed with IgM (SCCA-IgM). During the early phase of hepatocarcinogenesis, its expression is increased. Reports based on serum samples collected from HCC/cirrhosis patients and healthy volunteers, SCCA-IgM got a higher sensitivity value than AFP, but a lower specificity in HCC diagnosis. Therefore, SCCA-IgM may be an important serum biomarker for early detection of HCC [85].

### 5.2 Genetic and Epigenetic Events in HCC

HCC initiation and progression is associated with genetic alteration. The permanent genetic abnormalities build up in hepatocytes and cause disrupted gene expression which ultimately leads to cancerous transformation. Genetic alterations include large chromosomal translocation, amplification, single nucleotide variation, small fraction loss and deletion. The genetic changes frequently cause the loss of function or activation of oncogenes or tumour suppressor genes. Contrary to genetic alterations, no change in the genome sequence is found in epigenetic regulations but it influences the chromatin structure and transcription of the gene. Gene products are affected at transcriptional and post-transcriptional levels during epigenetic regulations which include DNA methylation, histone modification, and lncRNA. This provides greater diversity to the gene regulation [86].

#### 5.2.1 Chromosomal Instability

In HCC, chromosomal instability is the most frequently observed genetic changes. It could be promoted by either error during mitosis or disruption in DNA replication and repair processes. The chromosome abnormalities include amplification/deletion of small chromosomal segments or gain/loss of whole chromosome arms. Comparative genomic hybridization data in HCC represent frequent amplification of chromosome 1q and 8q, while the frequent loss of chromosome 1p, 4q, 6q, 9p, 16p, 16q, and 17p (Table **1**) [87]. Chromosome 1q amplification in HCC is a characteristic feature of chromosome abnormalities. In a large number of HCC patients, the chromosome 1q21 region containing CHD1L (an oncogene) was reported to be amplified [110]. CHD1L is associated with oncogenic functions during hepatocarcinogenesis such as anti-apoptotic, mitosis regulation, and stimulating cell epithelial-to-mesenchymal transition [88, 111]. In HCC chromosome, 8q24 region is another highly amplified region which contains oncogenes including c-Myc and PTK2 [98, 99, 112]. SGK3A, a serine/threonine kinase, having similarity with AKT is commonly amplified in HCC which provides AKT independent oncogenic roles [100]. Segmental loss of chromosome 1p35-36 region containing many tumour suppressors (14-3-3 σ and Rb-interacting zinc finger 1) is also commonly found in HCC. Short arm loss of chromosome 8 (a minimal region of 8p21-22 containing DLC-1) is a common feature in HCC. Because of promoter hypermethylation and allele loss, DLC-1 is recurrently deleted in HCC tissues [113]. DLC-1 expression restoration in hepatoma cells could induce cell apoptosis, and inhibit tumour growth [114].

#### 5.2.2 Circulating miRNAs

MicroRNA (miR), a class of non-coding RNAs, has been identified as important regulators of gene expression at post-transcriptional levels. Role of circulating miRNAs in serum as cancer biomarkers were described in 2008 and overexpression of miR-155, miR-21 and miR-210 were observed in B-cell lymphoma patients [115]. Abnormal expression of HCC development and progression related miRNAs and their role is under investigation. miR-122 and miR-221 regulate the cell cycle by modulating cyclins or cdk [116, 117]. Pro-apoptotic proteins (Bmf) are targets of some miRNAs (miR-221) which help HCC cells to avoid apoptosis [118]. However, some miRNA (miR29) can promote HCC apoptosis by targeting the Bcl-2 and Mcl-1, the anti-apoptotic proteins [119]. The most important characteristics of HCC *i.e.*, invasion and metastasis are also regulated by miRNAs. Cell migration and spreading in HCC is promoted by pro-metastatic miRNAs *e.g.*, miR-106b induces cell migration and invasion in HCC by activating epithelial-mesenchymal transition process [120]. Metastasis and HCC progression are suppressed by let-7g, miR-139, and miR-195 [121]. Unusually expressed miRNAs and their roles are given in Table **2**.

#### 5.2.3 Altered DNA Methylation Pattern

Abnormal DNA methylation is recurrently observed in human carcinomas. Methylation of cytosine residues in the promoter region takes place at CpG islands by DNA Methylase (DNMT). However, in tumour cells, the promoter methylation pattern is usually changed. Aberrant DNA methylation in the promoter regions of tumour suppressor genes results in transcriptional silencing and genomic instability by inhibiting the binding of RNA polymerase and transcription factors [86, 140, 141]. Hypermethylation is commonly observed at CpG islands in the promoter region of tumour suppressor genes in HCC. Suppressor of cytokine signalling, which regulates the JAK/STAT signalling pathway, was found to be silenced in more than 60% of HCC patients due to promoter hypermethylation [142]. It has been reported that multiple tumor-related genes, such as the APC, E-Cadherin and Hypermethylated-In-Cancer (HIC)-18 genes, are silenced by DNA hypermethylation in HCC [143, 144]. Stepwise increase in methylation of several genes was observed with the cancer progression. Upregulation of oncogenic signalling pathways such as JAK/STAT, Ras, and β-catenin/Wnt takes place by silencing tumour suppressors epigenetically in HCC has been revealed in genome-wide DNA methylation analysis [145].

## 6 Drug targets in HCC

### 6.1 Multikinase Inhibitors

Sorafenib (BAY43-9006, Nexavar**)** is multikinase inhibitor with dual inhibitory activity against RAF/MEK/ERK (Raf-1, B-Raf) in the tumour cell and vascular growth factor inhibitor family (VEGFR1, VEGFR2) and platelets derived growth factor receptor (PDGFR, c-Kit) which promote tumour progression and angiogenesis. Therefore, sorafenib acts either directly on the tumour or on angiogenesis and inhibits tumour growth [146]. Sunitinib malate (SU11248, Sutent; Pfizer, NY, USA) and Linifanib (ABT-869) are also oral multikinase inhibitors that act on growth factors and receptor tyrosine kinases involved in angiogenesis and HCC progression [147, 148].

### 6.2 Inhibitors of Mesenchymal-Epithelial Transition factor (MET) Receptor

C-MET is a protein, encoded by MET oncogene, possesses tyrosine kinase activity involved in tumour development and metastasis [149]. Tivantinib (ARQ 197) and cabozantinib are MET inhibitors that act by binding to its dephosphorylated state which is responsible for inhibition of growth and apoptosis in human tumour cell line [150, 151].

### 6.3 Angiogenesis Inhibitors

HCC is characterized by hyper vasculature resulting from higher expression of angiogenesis promoting factors viz., angiopoietin 2, PDGF, and VEGF [152]. Bevacizumab (Avastin; Genentech, CA, USA) is a humanised monoclonal antibody (mAb) acting on VEGF and one of the important drugs for colorectal cancer and liver metastasis of colorectal cancer [153]. Brivanib (BMS-582664), an inhibitor of VEGF and FGF signalling has shown efficacy as a first-line treatment for advanced HCC patients [154]. Ramucirumab (Cyramza), a mAb, is an inhibitor of VEGFR-2 [155].

### 6.4 PI3K/Akt/mTOR Inhibitors

Immunohistochemistry has shown that approximately 50% of HCC patients have activated mTOR pathway. This activation may be the result of increased signalling due to overexpression of ligands (EGF, IGF1, and IGF2) or may be due to mutant oncogenes (PI3KCA) or tumour suppressor genes (PTEN). Temsirolimus and Everolimus, an analogue of rapamycin are the inhibitors of mTOR [156].

## CONCLUSION

HCC is common and aggressive malignant tumour worldwide with a dreadful outcome. Multiple factors including viruses, chemicals as well as inborn and acquired metabolic diseases are responsible for its development. HBV and HCV are the major risk factors for virus-induced HCC development through direct or indirect mechanisms. HBV DNA integration into the host genome induces genomic instability and eventually directs insertional mutagenesis. Epigenetic changes targeting the expression of tumour suppressor genes also occur early in the development of HCC. Since HCC is a complex disease, therefore it is difficult to characterize HCC with a single biomarker. Several diagnostic markers including α-fetoprotein, des-γ-carboxyprothrombin, glypican-3, golgi protein-73, squamous cell carcinoma antigen, miRNAs and altered DNA methylation pattern are associated with HCC. Thus, the investigation on a combination of biomarker might provide valuable insight for diagnosis and prognosis. Sever drug classes acting on various targets like multikinase inhibitors, MET receptor inhibitor, angiogenesis inhibitors and mTOR inhibitors have shown efficacy in the treatment of HCC patients. Further researches on HCC are necessary to identify new biomarkers and drugs for early diagnosis and effective treatment.

## Figures and Tables

**Table 1:** Chromosomal aberration in hepatocellular carcinoma.

**Chromosome**	**Candidate target gene and their location**	**Aberration type**	**Reference**
**1q**	*CSK1B****(1q21.2)***	Gain	[88, 89]
	*CHD1L****(1q21.1)***		
	*JTB****(1q21)***		
	*MDM4****(1q32.1)***		
**1p**	*p18****(1p32)***	Loss	[90-92]
	*p73****(1p36.3)***		
	*RIZ****(1p36.13-p36.23)***		
**3q**	*Gankyrin ****(3q28)***	Gain	[33]
**3p**	*ROSSF1A****(3p21.3)***	Loss of heterogeneity, CpG methylation	[93, 94]
	*CTNNB1****(3p21)***		
	*TGF-1βR11****(3q22)***		
**4q**		Loss of heterogeneity	[95]
**6p**		Gain	[96]
**6q**	*M6P/IGF2R ****(6q26-q27)***	Loss of heterogeneity	[97]
**8q**	*c-Myc ****(8q24.21)***	Gain	[98.99,100]
	*PTK2 ****(8q24.3)***		
	*EIF3S3 ****(8q23.3)***		
	*SGK3 ****(8q13.1)***		
**8p**	*DLC-1 ****(8p21.3-22)***	Loss of heterogeneity, CpG methylation	[101]
	*LPTS ****(8p23)***		
	*CSMD1 ****(8p23.2)***		
**9p**	*CDKN2A ****(9p21)***	Loss of heterogeneity, CpG methylation	[102]
	*CDKN2B ****(9q21)***		
**11q**	*cyclinD1 (****11q13)***	Gain	[103]
**10q**	*PTEN/MMAC1 ****(10q23.3)***	Loss of heterogeneity	[104]
**11p**	*KAI1 ****(11p11.2)***	Loss of heterogeneity, CpG methylation	[105]
	*IGF-2 ****(11p15)***		
	*TSLC1 ****(11p23.2)***		
**13q**	*Rb1 ****(13q14.2)***	Loss of heterogeneity	[106, 107]
	*BRCA2 ****(13q12.3)***		
	*Tg737 ****(13q12.1)***		
	*TFDP1 ****(13q34)***		
	*CUL4A ****(13q34)***		
	*CDC1 ****(13q34)***		
**16q**	*CDH1 ****(16q22.1)***	Loss of heterogeneity, CpG methylation	[102]
**16p**	*Axin1 ****(16p13.3)***	CpG methylation	[108]
	*SOCS-1 ****(16p13.3)***		
**17p**	*p53 ****(17p13.1)***	Loss of heterogeneity	[103, 109]
	*HIC-1 ****(17p13.3)***		
	*HCCS1 ****(17p13.3)***		

**Table 2:** Aberrantly expressed miRNA and their reported target genes.

**S.N**	**miRNAs**	**Validated Gene targets**	**Function of miRNAs**	**Expression of miRNAs**	**References**
1	**miR-21**	PTEN, RECK, PDCD4	Anti-apoptotic activity, promotes metastasis and invasion	↑ ↑	[122, 123]
2.	**miR-106b**	E2F1, RhoGTPases, RhoA, RhoC	Promotes cell migration and actin stress fibre formation	↑ ↑	[120]
3.	**miR-17-5p**	p38, MAPK pathway, E2F-1, c-MYC	Promotes malignancy and metastasis	↑ ↑	[2, 124]
4.	**miR- 151**	RhoGDIA, FAK,	Promotes tumour metastasis and invasion	↑ ↑	[125, 126]
5.	**miR-122**	CyclinG1, ADAM10, SRF, IGF1R, PTTG1, PBF, CUTL1,NDRG3,MDR-1	Responsible for inhibition of virus replication and cell proliferation	↓ ↓	[127, 128]
6.	**miR-143**	FNDC3B	Promotes tumour metastasis	↑ ↑	[129]
7.	**miR-210**	VMP1	Promotes hypoxia induced epithelial to mesenchymal transition	↑ ↑	[130]
8.	**miR-29**	MEG3, Bcl-2, Mcl-1	Promotion of apoptosis and inhibition of tumour growth	↓ ↓	[119]
9.	**let-7**	cMyc, p16, Bcl-xl, COLIA2	Inhibit cell growth and proliferation	↓ ↓	[131]
10.	**miR-26a**	Cyclin D2, Cyclin E2, Cyclin E1,CDK6, IL-6	Inhibit metastasis, invasion and tumour growth	↓ ↓	[132]
11.	**miR-221**	CDKN1B/p27,CDKN1C/p57, DDIT4,PTEN, Bmf, TIMP3, PPP2R2A	Anti-apoptotic, help in metastasis and tumour growth.	↑ ↑	[118, 133]
12.	**miR-1**	FoxP1, MET, HDAC4	Inhibition of cell growth and reduced replication potential	↓ ↓	[134]
13.	**miR-195**	cyclin D1, CDK6, E2F3, LATS2,VEGF, VAV2, CDC42, IKKα and TAB3, TNF-α/NF-κB pathway	Inhibit metastasis, G1/S transition, angiogenesis and helps in apoptosis.	↓ ↓	[135]
14.	**miR-45**	OCT4, IRS1, IRS2, IGF signaling,HDAC2	Inhibit cell proliferation, migration, and invasion	↓ ↓	[136, 137]
15.	**miR-224**	API-5, CDC42, CDH1, PAK2, BCL-2,MAPK1, PPP2R1B. .	Promote cell proliferation,migration, invasion, andinhibit cell apoptosis	↑ ↑	[138, 139]
